# Supplementary material for: MetaRibo-Seq measures translation in microbiomes
Source: Nat Commun. 2020 Jun 29;11:3268. doi: 10.1038/s41467-020-17081-z (PMC7324362; doi:10.1038/s41467-020-17081-z)
Supplement: Supplementary file 10 — Supplementary Data 7 [file 41467_2020_17081_MOESM10_ESM.zip › File2/Confidence_High_Taxonomy/386460_out.krona.html]

Javascript must be enabled to view this page.

members
magnitude
magnitudeUnassigned
count
unassigned
taxon
rank

386460\_out

369

369
2
superkingdom

phylum
1239
368

class
368
186801

186802
368
order

family
1
186803

genus
1
841

species
301302
1

SRS142542\_contig\_number\_3310

2
216572
family

genus
2
459786

species
1
1945593

SRS143895\_contig\_number\_36709

1
876091
species

SRS143876\_contig\_number\_40454

541000
365
family

364
216851
1

SRS148874\_contig\_number\_2534
genus

species
2292357
1

SRS018351\_contig\_number\_contig-100\_7151.86810


SRS148721\_contig\_number\_42133
species
1
1262898

species
10
259315

SRS014948\_contig\_number\_contig-100\_3782.122283SRS018351\_contig\_number\_14218SRS019968\_contig\_number\_contig-100\_3095.100839SRS047014\_contig\_number\_38125SRS048164\_contig\_number\_contig-100\_2214.175325SRS077294\_contig\_number\_19556SRS098655\_contig\_number\_14125SRS105153\_contig\_number\_29072SRS142890\_contig\_number\_13049SRS143417\_contig\_number\_contig-100\_2568.180374

species
2302956
12

SRS013687\_contig\_number\_contig-100\_3499.175080SRS015782\_contig\_number\_45701SRS018313\_contig\_number\_contig-100\_5453.68212SRS049712\_contig\_number\_contig-100\_3935.121377SRS101433\_contig\_number\_8600SRS1055056\_contig\_number\_11516SRS143148\_contig\_number\_20505SRS143372\_contig\_number\_11578SRS147022\_contig\_number\_contig-100\_431.93965SRS147039\_contig\_number\_contig-100\_4628.30813SRS893270\_contig\_number\_contig-100\_4241.118250SRS971276\_contig\_number\_31134

species
2292356
2

SRS015579\_contig\_number\_788SRS148159\_contig\_number\_45360


SRS049995\_contig\_number\_contig-100\_1826.218892SRS104084\_contig\_number\_contig-100\_6316.69171SRS144183\_contig\_number\_30730SRS147977\_contig\_number\_3560SRS149879\_contig\_number\_13619
2302955
5
species


SRS014287\_contig\_number\_contig-100\_3732.109384SRS074670\_contig\_number\_contig-100\_1891.36270
1971605
2
species


SRS020869\_contig\_number\_contig-100\_2892.209894SRS045004\_contig\_number\_contig-100\_2446.183553SRS097920\_contig\_number\_24237SRS1041145\_contig\_number\_35878
4
2302957
species


SRS011084\_contig\_number\_17259SRS011239\_contig\_number\_27191SRS011302\_contig\_number\_16317SRS012273\_contig\_number\_47882SRS012849\_contig\_number\_33368SRS013098\_contig\_number\_34964SRS013638\_contig\_number\_contig-100\_973.124512SRS013687\_contig\_number\_contig-100\_3962.175543SRS013940\_contig\_number\_3460SRS013940\_contig\_number\_15733SRS013951\_contig\_number\_4486SRS013965\_contig\_number\_5196SRS013965\_contig\_number\_6707SRS014235\_contig\_number\_45695SRS014235\_contig\_number\_contig-100\_2183.263763SRS014287\_contig\_number\_3295SRS014313\_contig\_number\_contig-100\_1628.1628SRS014412\_contig\_number\_2918SRS014412\_contig\_number\_20940SRS014459\_contig\_number\_182SRS014459\_contig\_number\_36301SRS014613\_contig\_number\_16763SRS014683\_contig\_number\_contig-100\_2158.121786SRS014683\_contig\_number\_contig-100\_3257.122885SRS014923\_contig\_number\_contig-100\_6604.209519SRS014948\_contig\_number\_5165SRS014979\_contig\_number\_33937SRS015065\_contig\_number\_5195SRS015217\_contig\_number\_14168SRS015264\_contig\_number\_14535SRS015431\_contig\_number\_71089SRS015486\_contig\_number\_contig-100\_31.19873SRS015578\_contig\_number\_24054SRS015578\_contig\_number\_35861SRS015579\_contig\_number\_22459SRS015694\_contig\_number\_contig-100\_1183.139096SRS015782\_contig\_number\_7439SRS015782\_contig\_number\_contig-100\_3500.243307SRS015794\_contig\_number\_3363SRS015854\_contig\_number\_contig-100\_2093.137442SRS016056\_contig\_number\_4094SRS016132\_contig\_number\_771SRS016203\_contig\_number\_contig-100\_1975.71253SRS016335\_contig\_number\_contig-100\_971.260038SRS016495\_contig\_number\_12231SRS016629\_contig\_number\_contig-100\_2173.30042SRS016954\_contig\_number\_1390SRS016989\_contig\_number\_6352SRS017191\_contig\_number\_4054SRS017307\_contig\_number\_1379SRS017307\_contig\_number\_11500SRS017433\_contig\_number\_contig-100\_1891.102612SRS017859\_contig\_number\_4939SRS018427\_contig\_number\_16820SRS018575\_contig\_number\_contig-100\_1888.61355SRS018623\_contig\_number\_17934SRS018623\_contig\_number\_19753SRS018656\_contig\_number\_contig-100\_6215.68758SRS018836\_contig\_number\_15495SRS018836\_contig\_number\_19707SRS018888\_contig\_number\_1988SRS018936\_contig\_number\_contig-100\_1703.85407SRS018984\_contig\_number\_contig-80\_4471.172666SRS019030\_contig\_number\_14466SRS019068\_contig\_number\_107114SRS019161\_contig\_number\_26097SRS019397\_contig\_number\_11622SRS019445\_contig\_number\_3207SRS019445\_contig\_number\_25023SRS019496\_contig\_number\_17793SRS019496\_contig\_number\_contig-100\_1976.107346SRS019685\_contig\_number\_contig-100\_4583.199117SRS019693\_contig\_number\_8804SRS019693\_contig\_number\_contig-100\_1899.73820SRS019808\_contig\_number\_20942SRS019808\_contig\_number\_21037SRS019968\_contig\_number\_contig-100\_4190.101934SRS020233\_contig\_number\_43471SRS020328\_contig\_number\_17595SRS020328\_contig\_number\_27838SRS020328\_contig\_number\_37746SRS020394\_contig\_number\_17256SRS021484\_contig\_number\_23897SRS022071\_contig\_number\_15223SRS022137\_contig\_number\_contig-100\_13002.92264SRS022609\_contig\_number\_contig-100\_1108.270191SRS022609\_contig\_number\_contig-100\_1939.271022SRS023346\_contig\_number\_contig-100\_2080.2080SRS023526\_contig\_number\_34008SRS023715\_contig\_number\_contig-100\_3363.159725SRS024075\_contig\_number\_16856SRS024132\_contig\_number\_46692SRS024331\_contig\_number\_22968SRS024435\_contig\_number\_42364SRS024492\_contig\_number\_15651SRS024492\_contig\_number\_16261SRS024625\_contig\_number\_19400SRS024663\_contig\_number\_15915SRS042628\_contig\_number\_19083SRS042628\_contig\_number\_29901SRS042966\_contig\_number\_11871SRS043411\_contig\_number\_6641SRS043667\_contig\_number\_1068SRS043701\_contig\_number\_contig-100\_7206.97796SRS043768\_contig\_number\_31951SRS043841\_contig\_number\_9831SRS044535\_contig\_number\_contig-100\_8563.105172SRS045244\_contig\_number\_7164SRS045244\_contig\_number\_contig-100\_2160.61808SRS045739\_contig\_number\_11564SRS045826\_contig\_number\_23965SRS045826\_contig\_number\_23988SRS046068\_contig\_number\_2274SRS046369\_contig\_number\_15964SRS046502\_contig\_number\_3076SRS046502\_contig\_number\_3084SRS046717\_contig\_number\_6253SRS046717\_contig\_number\_contig-100\_752.86597SRS047014\_contig\_number\_29885SRS047044\_contig\_number\_3218SRS047433\_contig\_number\_24535SRS048060\_contig\_number\_9484SRS048870\_contig\_number\_19477SRS048981\_contig\_number\_8041SRS049446\_contig\_number\_7281SRS049446\_contig\_number\_contig-100\_1227.84176SRS049773\_contig\_number\_26246SRS049896\_contig\_number\_34043SRS049959\_contig\_number\_39529SRS049995\_contig\_number\_contig-100\_899.217965SRS050422\_contig\_number\_contig-100\_4869.172423SRS050752\_contig\_number\_3407SRS050752\_contig\_number\_contig-100\_3088.161898SRS050925\_contig\_number\_20155SRS050925\_contig\_number\_37859SRS050941\_contig\_number\_21498SRS050941\_contig\_number\_22366SRS051610\_contig\_number\_3172SRS051882\_contig\_number\_contig-100\_3148.120004SRS052027\_contig\_number\_contig-100\_4229.101303SRS052078\_contig\_number\_2718SRS052697\_contig\_number\_42582SRS053214\_contig\_number\_21535SRS053356\_contig\_number\_contig-100\_1666.263971SRS053356\_contig\_number\_contig-100\_2139.264444SRS053398\_contig\_number\_24320SRS054905\_contig\_number\_19947SRS054905\_contig\_number\_24375SRS054905\_contig\_number\_contig-100\_1417.155155SRS054956\_contig\_number\_4723SRS054956\_contig\_number\_13139SRS055017\_contig\_number\_34865SRS056273\_contig\_number\_contig-100\_3196.186032SRS056273\_contig\_number\_contig-100\_3730.186566SRS056519\_contig\_number\_contig-100\_7067.88130SRS057480\_contig\_number\_contig-100\_12571.12572SRS058770\_contig\_number\_24446SRS062427\_contig\_number\_contig-100\_5678.66910SRS062654\_contig\_number\_4533SRS063040\_contig\_number\_contig-100\_2839.258912SRS063190\_contig\_number\_contig-100\_1393.91419SRS063370\_contig\_number\_contig-100\_3105.72175SRS063518\_contig\_number\_15561SRS063985\_contig\_number\_29985SRS064276\_contig\_number\_1015SRS064276\_contig\_number\_24121SRS064276\_contig\_number\_37031SRS064757\_contig\_number\_11354SRS064757\_contig\_number\_23658SRS065397\_contig\_number\_contig-100\_1000.75610SRS065504\_contig\_number\_contig-100\_5908.172507SRS075021\_contig\_number\_contig-100\_176.60773SRS075078\_contig\_number\_23471SRS075078\_contig\_number\_contig-100\_3097.184640SRS075078\_contig\_number\_contig-100\_3140.184683SRS075341\_contig\_number\_24960SRS075773\_contig\_number\_270SRS075773\_contig\_number\_32505SRS075821\_contig\_number\_24384SRS075984\_contig\_number\_20975SRS076804\_contig\_number\_2185SRS076804\_contig\_number\_15930SRS076976\_contig\_number\_946SRS076976\_contig\_number\_16625SRS077024\_contig\_number\_14218SRS077127\_contig\_number\_11819SRS077194\_contig\_number\_10689SRS077194\_contig\_number\_22859SRS077231\_contig\_number\_24535SRS077231\_contig\_number\_contig-100\_4032.133755SRS077392\_contig\_number\_44013SRS077454\_contig\_number\_3433SRS077454\_contig\_number\_17696SRS077454\_contig\_number\_contig-100\_8601.100151SRS077502\_contig\_number\_616SRS077552\_contig\_number\_contig-100\_1172.172418SRS077589\_contig\_number\_contig-100\_3728.3729SRS077730\_contig\_number\_contig-100\_2384.93241SRS077849\_contig\_number\_32262SRS078176\_contig\_number\_contig-100\_765.143290SRS078242\_contig\_number\_contig-100\_2016.194146SRS078242\_contig\_number\_contig-100\_6574.198704SRS097889\_contig\_number\_1307SRS097889\_contig\_number\_14095SRS097920\_contig\_number\_7604SRS097958\_contig\_number\_8756SRS097958\_contig\_number\_18117SRS097958\_contig\_number\_contig-100\_6097.118484SRS098061\_contig\_number\_13028SRS098073\_contig\_number\_18283SRS098571\_contig\_number\_55225SRS098571\_contig\_number\_64775SRS098644\_contig\_number\_2243SRS098644\_contig\_number\_3784SRS098881\_contig\_number\_19199SRS100021\_contig\_number\_12386SRS101376\_contig\_number\_22620SRS101376\_contig\_number\_27405SRS103987\_contig\_number\_33823SRS104084\_contig\_number\_contig-100\_7184.70039SRS1041036\_contig\_number\_4862SRS1041037\_contig\_number\_6972SRS1041037\_contig\_number\_contig-100\_4727.78499SRS1041039\_contig\_number\_28218SRS1041090\_contig\_number\_contig-100\_176.50243SRS1041091\_contig\_number\_21116SRS1041118\_contig\_number\_18028SRS1041129\_contig\_number\_12410SRS1041134\_contig\_number\_12351SRS1041137\_contig\_number\_contig-100\_5563.145340SRS1041141\_contig\_number\_10164SRS1041157\_contig\_number\_contig-100\_1776.53098SRS104311\_contig\_number\_41SRS104311\_contig\_number\_1558SRS104400\_contig\_number\_25242SRS104400\_contig\_number\_47526SRS104485\_contig\_number\_1800SRS104636\_contig\_number\_contig-100\_1304.159455SRS104693\_contig\_number\_3102SRS104912\_contig\_number\_16668SRS104912\_contig\_number\_22078SRS104975\_contig\_number\_contig-100\_2668.123437SRS1055034\_contig\_number\_contig-100\_2560.65068SRS1055043\_contig\_number\_24972SRS1055049\_contig\_number\_17079SRS1055067\_contig\_number\_16236SRS1055069\_contig\_number\_2366SRS1055099\_contig\_number\_contig-100\_1784.87434SRS140492\_contig\_number\_13497SRS142890\_contig\_number\_7245SRS142890\_contig\_number\_41371SRS142923\_contig\_number\_22489SRS142923\_contig\_number\_22822SRS142980\_contig\_number\_5863SRS143148\_contig\_number\_934SRS143181\_contig\_number\_12860SRS143181\_contig\_number\_14287SRS143342\_contig\_number\_20948SRS143372\_contig\_number\_11886SRS143466\_contig\_number\_203SRS143466\_contig\_number\_7844SRS143466\_contig\_number\_29634SRS143523\_contig\_number\_23871SRS143523\_contig\_number\_25965SRS143722\_contig\_number\_contig-100\_678.94145SRS143895\_contig\_number\_910SRS143991\_contig\_number\_27888SRS144135\_contig\_number\_2988SRS144135\_contig\_number\_22428SRS144135\_contig\_number\_33432SRS144183\_contig\_number\_2263SRS144362\_contig\_number\_6477SRS144362\_contig\_number\_17183SRS144506\_contig\_number\_2923SRS144506\_contig\_number\_2944SRS144506\_contig\_number\_3050SRS144714\_contig\_number\_3202SRS144714\_contig\_number\_7556SRS144714\_contig\_number\_10751SRS146764\_contig\_number\_24164SRS146812\_contig\_number\_46190SRS146812\_contig\_number\_contig-100\_12198.231075SRS146813\_contig\_number\_26630SRS147139\_contig\_number\_15122SRS147271\_contig\_number\_contig-100\_9802.155742SRS147346\_contig\_number\_12737SRS147377\_contig\_number\_11137SRS147377\_contig\_number\_11800SRS147425\_contig\_number\_9859SRS147557\_contig\_number\_30652SRS147614\_contig\_number\_10829SRS147653\_contig\_number\_6943SRS147919\_contig\_number\_577SRS147919\_contig\_number\_14442SRS147977\_contig\_number\_348SRS148091\_contig\_number\_10730SRS148091\_contig\_number\_14719SRS148196\_contig\_number\_41672SRS148253\_contig\_number\_10329SRS148319\_contig\_number\_7659SRS148319\_contig\_number\_17188SRS148424\_contig\_number\_33312SRS148424\_contig\_number\_34871SRS148721\_contig\_number\_724SRS148784\_contig\_number\_contig-100\_4159.149783SRS148817\_contig\_number\_11716SRS149075\_contig\_number\_321SRS149075\_contig\_number\_2258SRS149325\_contig\_number\_contig-100\_2551.105286SRS149784\_contig\_number\_13828SRS149784\_contig\_number\_contig-100\_6157.137277SRS150029\_contig\_number\_20483SRS892882\_contig\_number\_11172SRS893259\_contig\_number\_contig-100\_345.54176SRS893270\_contig\_number\_23597SRS893270\_contig\_number\_24124SRS893292\_contig\_number\_6636SRS893295\_contig\_number\_11024SRS893300\_contig\_number\_contig-100\_5438.41903SRS893327\_contig\_number\_contig-100\_1084.72966SRS893358\_contig\_number\_contig-100\_3111.68908SRS893373\_contig\_number\_4300SRS893378\_contig\_number\_1452SRS893378\_contig\_number\_23299SRS971275\_contig\_number\_43978SRS971276\_contig\_number\_36296
species
853
326


SRS023914\_contig\_number\_22016
1898205
1
species

1
201174
phylum

84998
1
class

order
1
84999

1
84107
family

genus
102106
1


SRS098717\_contig\_number\_15742
species
1
626935
